# Osteoporosis Is Characterized by Altered Expression of Exosomal Long Non-coding RNAs

**DOI:** 10.3389/fgene.2020.566959

**Published:** 2020-11-12

**Authors:** Zhaowei Teng, Yun Zhu, Xiguang Zhang, Yirong Teng, Sheng Lu

**Affiliations:** ^1^The 6th Affiliated Hospital of Kunming Medical University, Yuxi, China; ^2^Yunnan Key Laboratory of Digital Orthopedics, The First People's Hospital of Yunnan Province, Kunming, China; ^3^The 920 Affiliated Hospital of Kunming Medical University, Kunming, China

**Keywords:** osteoporosis, serum exosomes, patient serum derived exosomal lncRNAs, diagnostic markers, therapeutic modules

## Abstract

Osteoporosis is a metabolic bone disease characterized by a decrease in bone mass and degradation of the bone microstructure, which increases bone fragility and risk of fracture. However, the molecular mechanisms of osteoporosis remain unclear. The current study attempts to elucidate the role of exosomal long non-coding RNA in the pathology of osteoporosis. Peripheral blood was collected from persons with (OP) or without (NC) osteoporosis, and the serum exosomes were extracted using ultra centrifugation process. Total RNA of exosomes was isolated, and the lncRNAs profiling was done using RNA-Seq experiments. *In silico* analysis resulted in identification of 393 differentially expressed (DE) lncRNAs in OP vs. NC, with 296 that were up-regulated and 97 were down-regulated. Bioinformatics analysis of potential target mRNAs of lncRNAs with cis-acting mechanism showed that mRNAs co-located with DE lncRNAs were highly enriched in osteoporosis-related pathways, including regulation of insulin secretion, activation of MAPK activity, cellular response to metal ions, fucosylation and proteolysis. Together these results suggest that lncRNAs of serum exosomes could play a significant role in development of osteoporosis and such information may be helpful in developing diagnostic markers and therapeutic modules for osteoporosis.

## Introduction

Bone remodeling is a complex process that is characterized by an equilibrium between bone loss and bone formation through which the bone strength is maintained (Zhao et al., 2018). Imbalance in this equilibrium process results in loss of bone mass thereby increasing the risk of fracture due to bone fragility. The incidence of loss of bone mass-associated fracture is high across the globe and positively correlated with age (Xie et al., 2018). This loss in bone mass with age due to disequilibrium of/imbalance in remodeling causing bone resorption is termed as osteoporosis and is a major cause for bone fracture in geriatric population.

Bone remodeling is a continuous process through which bone maturation takes place. This is mainly coordinated by two cell types in bone tissue called osteocytes and osteoclasts. The osteoclast cells are responsible for bone resorption or bone loss while osteoblasts promote ossification or bone formation (Behera and Tyagi, 2018). This mechanism is also activated in case of bone damage and repair mechanisms to restore bone mass. In case of patients with osteoporosis and osteoarthritis conditions, the bone reformation process is highly delayed as the inherent potential for bone reformation is low and osteoclast cell activity is high, contributing to reduced bone density (Henriksen et al., 2014; Tella and Gallagher, 2014). The process of bone remodeling coordinated by the bone resorbing osteoclast and bone forming osteoblasts is mediated by a variety of signaling processes including cell- cell signaling and other secretory signaling molecules including cytokines (Sims and Walsh, 2012). Recent studies have indicated that exosomes also play an important role in regulatory signaling of bone remodeling process. “Exosomes are defined as cell-derived vesicles formed from invagination of the cell membrane released to the extracellular space against different stimuli” (Carretero-González et al., 2018). Exosomes are small vesicles of 40–100 nm in size that were identified to play the role of signaling mediators in intra-cellular communications. Various studies have emphasized on the role of exosomes in regulating and coordinating bone remodeling especially in osteoporotic conditions (Sun et al., 2016; Xie et al., 2017). Thus, it becomes crucial to identify the change in exosome associated signaling mechanisms in pathological conditions like osteoporosis.

Exosomes from bone cells released to the bone micro-environment are shown to regulate various signaling cascades associated with bone cell proliferation and differentiation through paracrine or endocrine signaling mechanism (Sun et al., 2016). Exosomes secreted by osteoblasts and osteoclasts play a crucial role in coordinating bone remodeling process (Li et al., 2016). Recent reports suggest that exosomes coordinate signaling cascades including transcriptional regulation through non-coding RNAs like microRNAs and long non-coding RNAs (Cao et al., 2019). Here we emphasize on exploring the role of exosomal lncRNA in regulation and expression of osteoporosis related genes. lncRNAs are reported to act as both osteogenic differentiation inhibitors and promotors. They are also associated with pathologic conditions of bone cells like osteoporosis, osteosarcoma and in cellular process like differentiation of osteoblasts (Wu et al., 2018; Silva et al., 2019). High throughput screening technology have identified long non-coding RNAs of around 200 nt long in human transcriptomes. lncRNAs are also said to modify osteo- associated gene expression through transcriptional and post transcriptional regulation by genetic and epigenetic modifications. IncRNAs regulate gene expression by recruiting transcriptional factors, by acting on the RNA polymerase machinery or by sequestrating the microRNA that silence the mRNA expression (Dykes and Emanueli, 2017; Silva et al., 2019). A study by Liu et al. (2019), reported that about 1,500 lncRNAs were differentially expressed in case of osteoporotic conditions out of which around 250 lncRNAs showed significant variation. Other studies also have indicated the roles of few long non-coding RNAs like lncRNA RP11-498C9, lncRNA XR111050, lncRNA ANCR in regulation of osteogenic gene expression (Li et al., 2019; Silva et al., 2019). These observations lead to explore and identify the role of lncRNAs and their association with osteogenic gene expression.

This study critically looks at differential expression of various exosomal lncRNAs in osteoporotic conditions in comparison with those in normal condition. Serum derived exosomes (SDEs) were extracted from osteoporotic patients and were compared with SDEs from normal healthy individuals as control to identify the role of exosomal lncRNAs in osteoporotic conditions. Differentially expressed (DE) lncRNA target genes found by co-location analysis were associated with osteoporosis-related pathways, implying a crucial role of these lncRNAs in the disease. This will aid in exploring the mechanism of regulation in bone remodeling and the pathogenesis of bone loss. The study may also help in identifying new treatment methodologies including development of diagnostic biomarkers for detection of osteoporosis.

## Materials and Methods

### Patients and Clinical Samples

We collected serum from 9 elderly patients with fracture and 9 age-matched patients without fracture at the age between 60 and 90 years old from the Yuxi hospital, China. Either elderly postmenopausal women with low-energy fractures (that is, osteoporotic fractures) or age-matched women undergone revision total hip/knee arthroplasty without fracture history were included (inclusive criteria). Patients with diabetes, malignancy, coronary heart disease, or other severe diseases in the previous 5 years were excluded in our study (exclusive criteria). The Committees of Clinical Ethics of the three hospitals approved the sample collection procedures, which conformed to the principles of the Helsinki Declaration. We also obtained informed consent from the participants.

For each set three samples were mixed and that is considered as a one biological replicate. Three patients subjects were enrolled for testing and validation studies, respectively, between January 2018 and July 2018, as well as three healthy individuals matched for sex and age in the testing and validation sets, respectively.

The plasma samples of the study participants were collected in vacuum blood tubes with anticoagulant before operation and pharmacotherapy and handled within 1 h after collection. All individuals provided their written consent for using their plasma samples and pathologic information for the purpose of this research. The studies were conducted in accordance with the International Ethical Guidelines for Biomedical Research Involving Human Subjects (CIOMS), and protocol was reviewed and approved by the Research Ethics Committee of the First Affiliated Hospital of Kunming Medical University.

### Plasma Exosome Isolation

Blood samples were centrifuged at 1,500 g for 15 min at 4°C. The collected supernatant (2.5 mL plasma) were then centrifuged at 12,000 g for 30 min at 4°C. The supernatant was collected and ultra-centrifuged at 100,000 g for 2 h at 4°C to pellet the exosomes. Exosome pellets were then washed in filtered PBS and re-centrifuged at 100,000 g, the supernatant was removed and the final exosomal pellet was re-suspended in 200 μl PBS. The size distribution of exosomes was examined using a NanoSight Tracking Analysis LM20 System (NanoSight Ltd).

### Nanoparticle Tracking Analysis

Supernatants containing vesicles were analyzed using a NanoSight LM20 instrument equipped with a 640 nm laser (NanoSight, Amesbury, UK) at 25°C. Particle movement was tracked by NTA software (version 2.2, NanoSight) with low refractive index corresponding to cell-derived vesicles. Each track was then analyzed to get the mean, mode, and median vesicle size together with the vesicle concentration (in millions) for each size.

### RNA Extraction and Library Construction

Total RNA in exosomes were isolated using TRIzol (Invitrogen) reagent as per manufacturer's instructions. To remove DNA, the RNA was treated with RQ1 DNase (Promega, Madison, WI, USA) for 20 min at 37°C. The quality and quantity of the purified RNA were confirmed by measuring the absorbance at 260 and 280 nm (A 260 and A 280) using a SmartSpec Plus Spectrophotometer (Bio-Rad Laboratories, Inc., Hercules, CA, USA). RNA integrity was further verified by electrophoresis using a 1.5% agarose gel. All RNA samples were stored at −80°C for future use.

Purified mRNA was fragmented at 95°C followed by end repair and 5′ adaptor ligation. Reverse transcription was then performed with RT primers harboring 3′ adaptor sequences and randomized hexamers. cDNA was purified and amplified, and 200–500 bp PCR products were purified, quantified, and stored at −80°C until sequencing. cDNA clusters were generated and sequenced using an Illumina HiSeq2000 platform following the manufacturer's instructions to obtain 50-nt pair-end sequence reads. Two lanes were used for sequencing and Samples were separated by barcodes.

### LncRNA Identification and Classification

Raw reads generated by sequencing were subjected to several quality checks. The low quality reads were removed by read trimming and read filtering. Read trimming included removal of adapter sequences, removal of the 30 ends with low quality scores and trimming based on High-Residual Ionogram Values. Filtering of entire reads included removal of short reads, adapter dimers, reads lacking sequencing key, reads with off-scale signal and polyclonal reads. Subsequent analysis was performed with high quality reads which passed through the described above filtering steps.

High quality clean reads were selected for downstream analyses by filtering out low quality reads and adapters. These high quality reads were then mapped to the human genome using HISAT2 software. Only mapped reads with one genomic location were used for analysis. Fragments Per Kilobase Million (FPKM) method was used to evaluate the expression level of genes and lncRNAs.

In order to evaluate the pattern of lncRNA expression and screen DE lncRNAs, the R package (Robinson et al., 2010) which is specifically designed to analyze differentially expressed (DE) genes using RNA-seq data, was applied. To explore DE lncRNAs, fold change (FC) (≥2 or ≤0.5) and false discovery rate (FDR) (≤ 0.01) cutoffs were used.

The reads were assembled by Cufflinks and the assembled transcripts were merged together using the Cuffmerge procedure for downstream analysis. Cufflinks and Cuffcompare was utilized to identify novel transcripts. Background noise was removed from novel transcripts based on the following parameters: FPKM (>0.5), length (>200), coverage (>1), and status threshold (OK). The coding potential was determined using the Coding Potential Calculator (value < 0). StringTie software used the results of HISAT2 to compute transcription splicing.

### Gene Ontology Analysis

After screening specific genes according to the purpose of the experiment, the distribution of specific genes in the Gene Ontology will be studied to clarify the expression of sample differences in Gene function in the experiment. GO enrichment analysis method is GOseq (Young et al., 2010), which is based on the Wallenius non-central hyper-geometric distribution. Compared to ordinary hypergeometric distributions, the characteristic of this distribution is that the probability of extracting an GO term from a category is different from that of extracting an GO term from outside a category. The difference of this probability is obtained by estimating the bias of gene length, so as to more accurately calculate the probability that GO term is enriched by different genes.

### Identification of Common and Specific LncRNAs

Based on the transcriptome splicing results, a series of strict screening conditions were set according to the structural characteristics of lncRNAs and the functional characteristics of non-coding proteins. The transcriptome gtf files generated were merged to a single gtf file using Cuffmerge. These merged gtf files were used to analyze the loci of lncRNAs from different sequencing samples using Cuffcompare. Briefly, the steps are as follows; Step 1- Selecting transcripts with ≥2 Exons, Step 2- The transcript length of >200 bp was selected, Step 3- lncRNAs that overlap with the exon region of the splicing transcript in the database were included, Step 4- the Cuffquant expression of each transcript of FPKM ≥0.5 was selected, Step 5- Coding potential screening by coding potential calculator and Pfam-scan tools.

### Expression and Functional Analysis

The expression levels of lncRNA and mRNA were compared and analyzed to obtain the differences in the overall expression levels of different transcripts. The specific method was used to average the expression levels of each transcript among different samples. For repeated samples under the same experimental conditions, the final FPKM is the average of all repeated data.

### The Exploration of DE LncRNAs

The differentially expressed lncRNAs and mRNAs were further explored by three methods. The volcano map was used to visualize the overall distribution of different transcripts or genes, and setting the threshold *q* < 0.05. The distribution of DE lncRNAs and mRNAs on the chromosomes was analyzed to understand the relation between lncRNAs and mRNAs. Finally, hierarchical clustering analysis was done to group the differentially expressed transcripts into closely related clusters or classes. The FPKM value of the differential transcript under different experimental conditions was used as the expression level for hierarchical clustering analysis.

### LncRNA Targeted mRNA Prediction

The differentially expressed lncRNA and the co-located and co-expressed mRNA genes were analyzed by GO, KEGG enrichment analysis and protein network interaction analysis, respectively. For mRNA prediction of lncRNA co-location, a threshold of co-location as 100 kb upstream and downstream of lncRNAs was set. Subsequently, function enrichment analysis was performed on mRNA genes of lncRNA co-location to predict the main function of lncRNAs. Since the samples were <25, Pearson correlation coefficient method was used to analyze the correlation between lncRNA and mRNA among samples, and mRNA genes with the absolute value of correlation >0.95 were selected for functional enrichment analysis to predict the main function of lncRNA.

### cDNA Synthesis and Quantitative Real-Time PCR (qRT-PCR)

Reverse transcription reactions were performed using 3 μg of total RNA at 50°C in a 1 h reaction. The reaction mix comprised of 20 U RNase inhibitor (Invitrogen, Carlsbad, CA, USA), 200 U superscript II (Invitrogen), 500 ng random hexamers, and RT buffer.

The qRT-PCR was performed on a Bio-Rad S1000 with Bestar SYBR Green RT-PCR Master Mix (Toyobo). All the primers used in the study are available in the Table 1. PCR conditions included denaturing at 95°C for 1 min, and 40 cycles of denaturing at 95°C for 15 s followed by annealing and extension at 60°C for 30 s. Relative gene expression was calculated using the Livak and Schmittgen 2^−ΔΔ*Ct*^ method (Livak and Schmittgen, 2001), normalized with the reference gene Actin. PCR amplifications were performed in triplicate for each sample.

**Table 1:** List of primers used in the study.

lnc000727-F	CAGCTCGTTCATGGATGCAA
lnc000727-R	GAGAGATGTCATTGCCGCAG
lnc016368-F	TATTCCGAACCCAAGCACCT
lnc016368-R	TTTTGACACCAGCAGCATCC
lnc016350-F	ACTCCCAATCAGTCTGCCAT
lnc016350-R	TTCACGGCTCTCCATCAGTT
lnc016907-F	TTCACGGCTCTCCATCAGTT
lnc016907-R	GACACCCATATCCACGGACA
lnc003660-F	TAAGGAGGCTGCTTCACTGGAG
lnc003660-R	CTCTGAACCACCTTTGTCTCTGC
lnc005250-F	ATGCCATTGACCCGACTTGC
lnc005250-R	GAACCTCTGTCTGTGGATTCTGTG
lnc021013-F	GCCCGTCTCATCCCTTCTGG
lnc021013-R	TCTACCTGCCTTGTCCCTGTTC
lnc000682-F	GCAAGACAAGGGATTAAACCAAGG
lnc000682-R	CAACAACATCTGAGGAAGCACAAC
lnc013022-F	TGTTTCCCATTGTTATGCCTCCAG
lnc013022-R	CAACTGTGGTCACTCAACTGCTAG
lnc012605-F	CCATTCCTTCTGCTCAGGTGTC
lnc012605-R	ACCAGAGATGCCTTTCCCAGAG
lnc012972-F	CTCAGCAGTCCGAGGAACAATG
lnc012972-R	GCAGCAGAATTATCAAAGGAGACC
Actin-F	TGGACTTCGAGCAAGAGATG
Actin-R	GAAGGAAGGCTGGAAGAGTG

*Specific primers were designed according to lncRNA sequences selected from the differentially expressed lncRNAs*.

## Results

### Characterization of Serum Derived Exosomes

**Figure 1:**
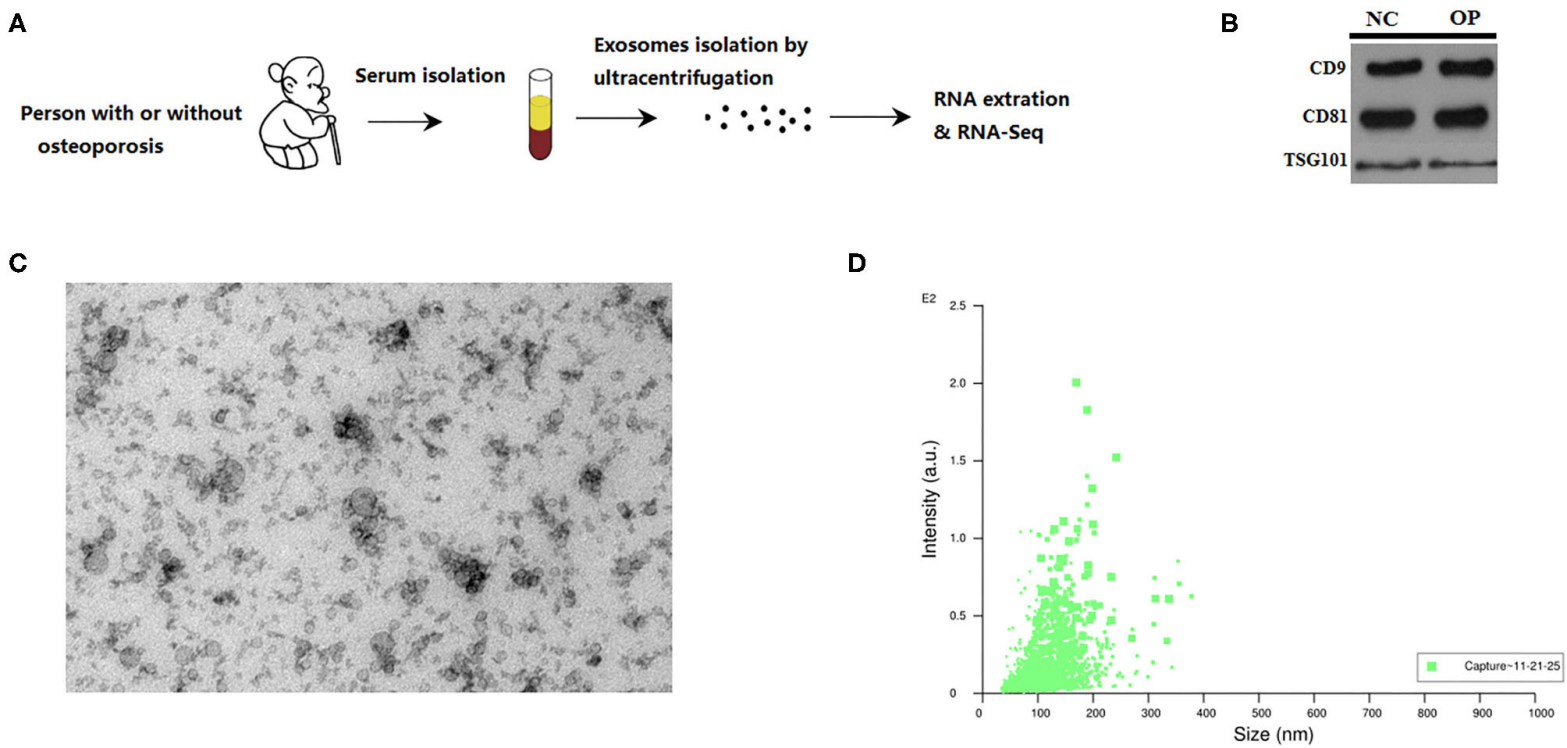
Characterization of serum exosomes from persons with or without osteoporosis and fracture. **(A)** A schematic diagram illustrating the experimental design from sample collection till sequencing. **(B)** Exosome validation by western blot indicating the CD9 and CD81 marker for exosomes. **(C)** TEM image of the exosomes. Electron microscopy allowed visualizing membrane-bound nano vesicles sized ~100 nm. Scale bar = 100 nm. **(D)** Analysis of exosomes with NanoSight LM10-HS instrument.

A simple process for isolating extracellular vesicles (EVs) from serum of individuals followed by evaluating their functional role in the pathophysiology of osteoporosis was demonstrated graphically in Figure 1A and described in detail in materials and methods section.

Prior to RNA sequencing EVs are characterized for their uniform size and by western blot. We run the protein blots and probe for exosome marker like CD9 and CD81 (Figure 1B). RNA extracted from the isolated extracellular vesicles were subjected to high throughput sequencing. EVs are known to come in various sizes and with different structural composition, hence, the EVs were characterized through electron microscopy and nanoparticle tracking. Transmission electron micrographs depicted membrane-bound EVs, also called as exosomes, with an average size of 100 nm (Figure 1C). These observations were then corroborated by performing another technique called nanoparticle tracking analysis based on the Brownian motion of particles in liquid that relates to the size of the particle. These observations suggest that the particle size ranges between 20 nm to 200 nm with the maximum intensity for particles of ~100 nm size (Figure 1D). Thus, we could obtain exosomes of ~100 nm in size from the serum of individuals that were analyzed further.

### High Throughput Analysis of LncRNAs From Exosomes

**Figure 2:**
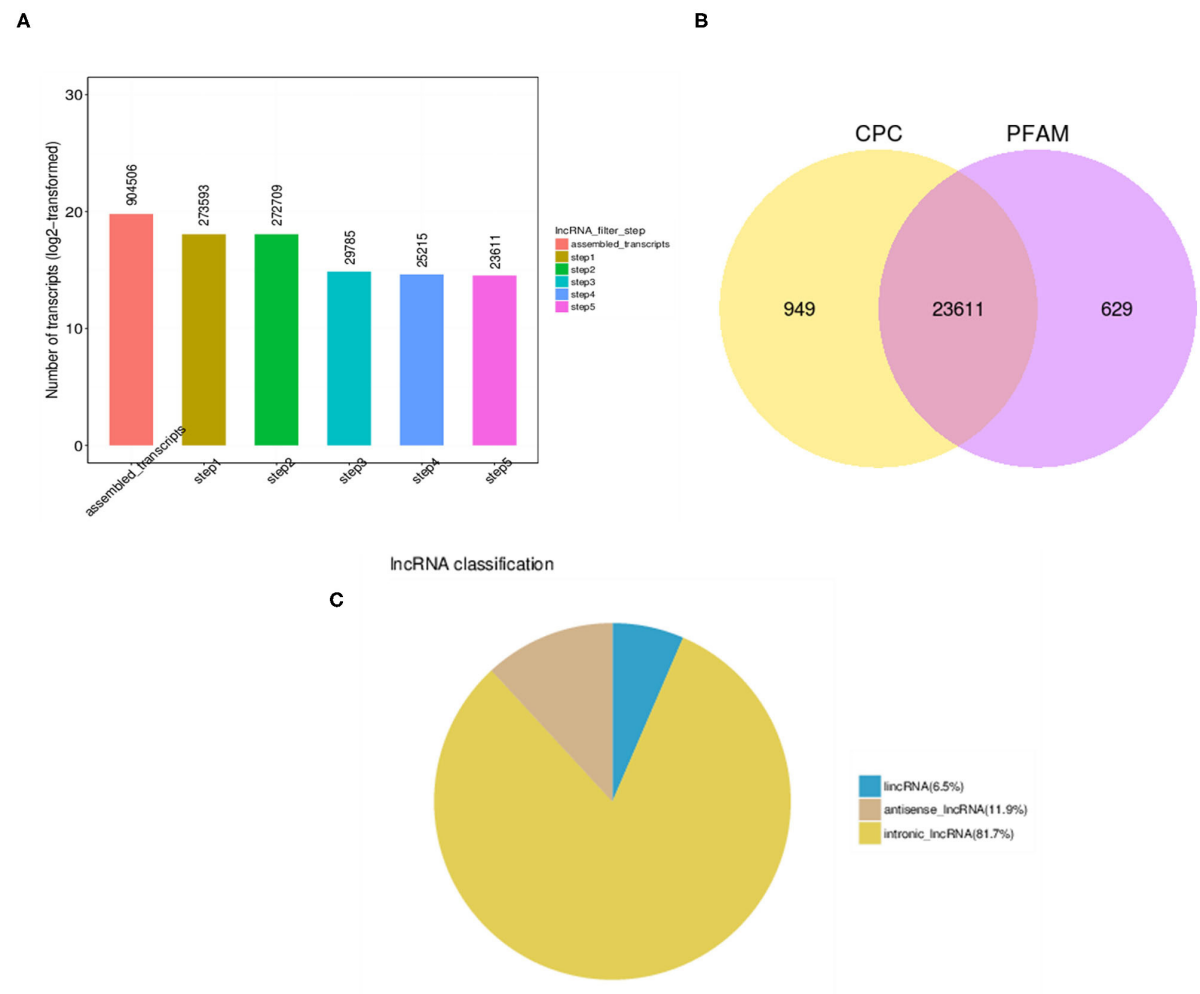
LncRNA identification analysis. **(A)** High throughput analysis steps indicating the number of transcripts obtained after each filtration step. **(B)** Number of overlapping candidate transcripts indicated by Venn diagram of coding potential analysis by Coding Potential Calculator (CPC) and Protein family (PFAM) tools. **(C)** Pie chart of different types of lncRNAs indicating that major lncRNA are from intronic region.

EVs isolated from serum as membrane-bound, 100 nm exosomes were then utilized to extract lncRNAs and to evaluate the association of these exosomal lncRNAs with osteoporosis. Toward that, total RNA extracted from exosomes from both healthy (NC) and osteoporotic patients (OP) was subjected to RNA-sequencing. High throughput long non-coding RNA analysis and mRNA splicing analysis could help identify both mRNAs and lncRNAs. A total of 904,506 transcripts were assembled, which were then eventually filtered down to 23,611 lncRNA transcripts for final analysis (Figure 2A) according to steps mentioned in the materials and methods. These lncRNAs transcripts were seen as common among the two coding potential tools (Figure 2B) of which 11.9% were antisense lncRNAs and 81.7% were intronic lncRNAs (Figure 2C).

### Comparison of Structural Features Between mRNA and LncRNAs in Osteoporosis

**Figure 3:**
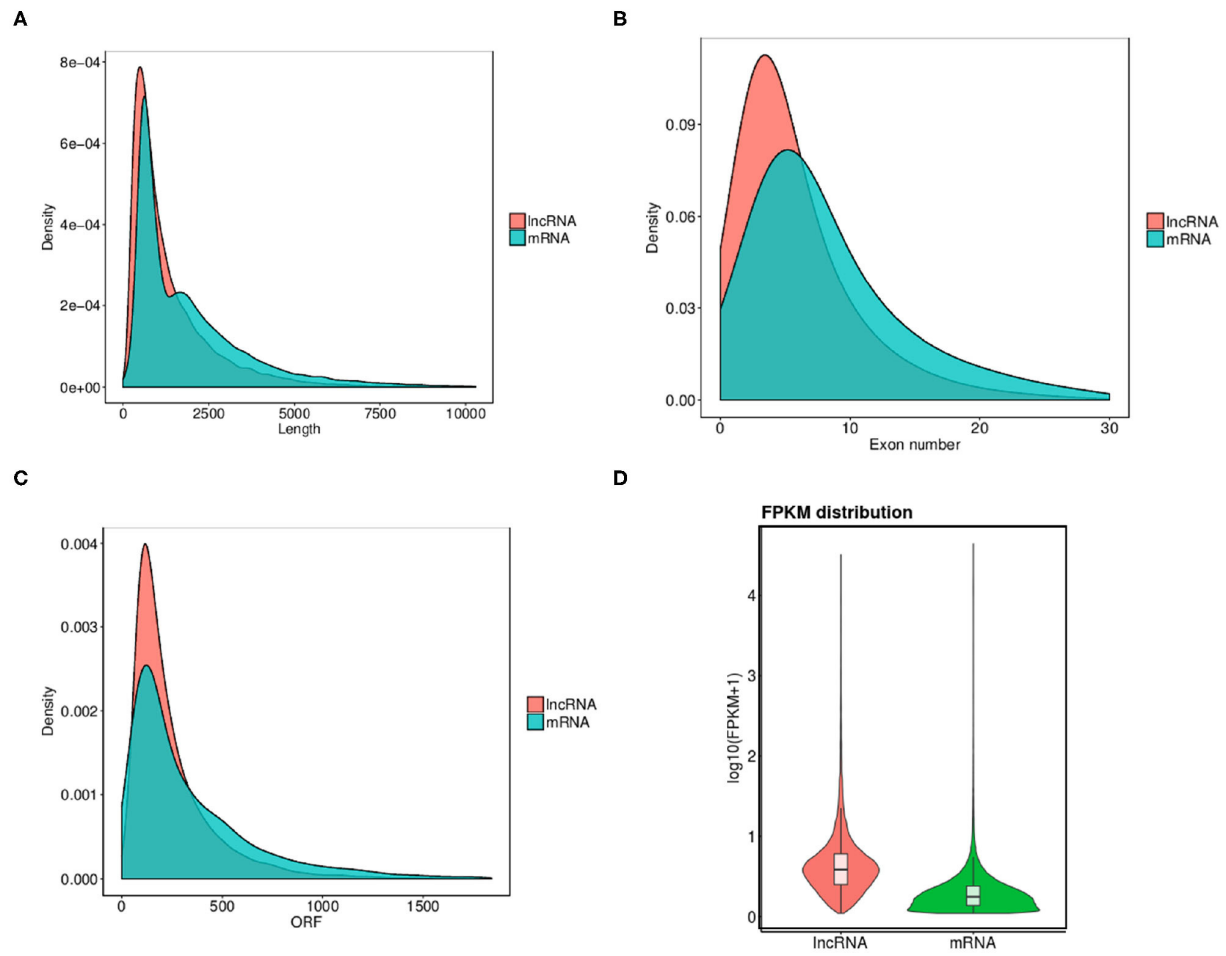
Comparison of structural features between mRNA and lncRNAs in osteoporosis. **(A–C)** Comparison of transcript length, exon number and ORF length distribution of transcripts between lncRNAs and mRNAs. **(D)** Overall expression levels of different transcripts of mRNAs and lncRNAs.

In order to completely understand the characteristic nature of exosomal lncRNAs and their association with mRNAs, a combination of structural features were analyzed between the two (Figure 3). Osteoporosis associated lncRNAs were shorter in length as compared to the mRNA's detected from the patients samples (Figure 3A). The exon number and ORF length of lncRNAs were also lesser as compared to that of mRNAs (Figures 3B,C). The expression difference of all transcripts of lncRNAs and mRNAs was studied through the FPKM distribution analysis (Figure 3D). The results showed that overall expression difference of mRNAs was relatively significant and most of mRNAs were usually expressed at low levels while the expression alterations of lncRNAs were uniform between the maximum and least FPKMs (Figure 3D).

### Expression Analysis of Exosome Associated LncRNAs and mRNAs

**Figure 4:**
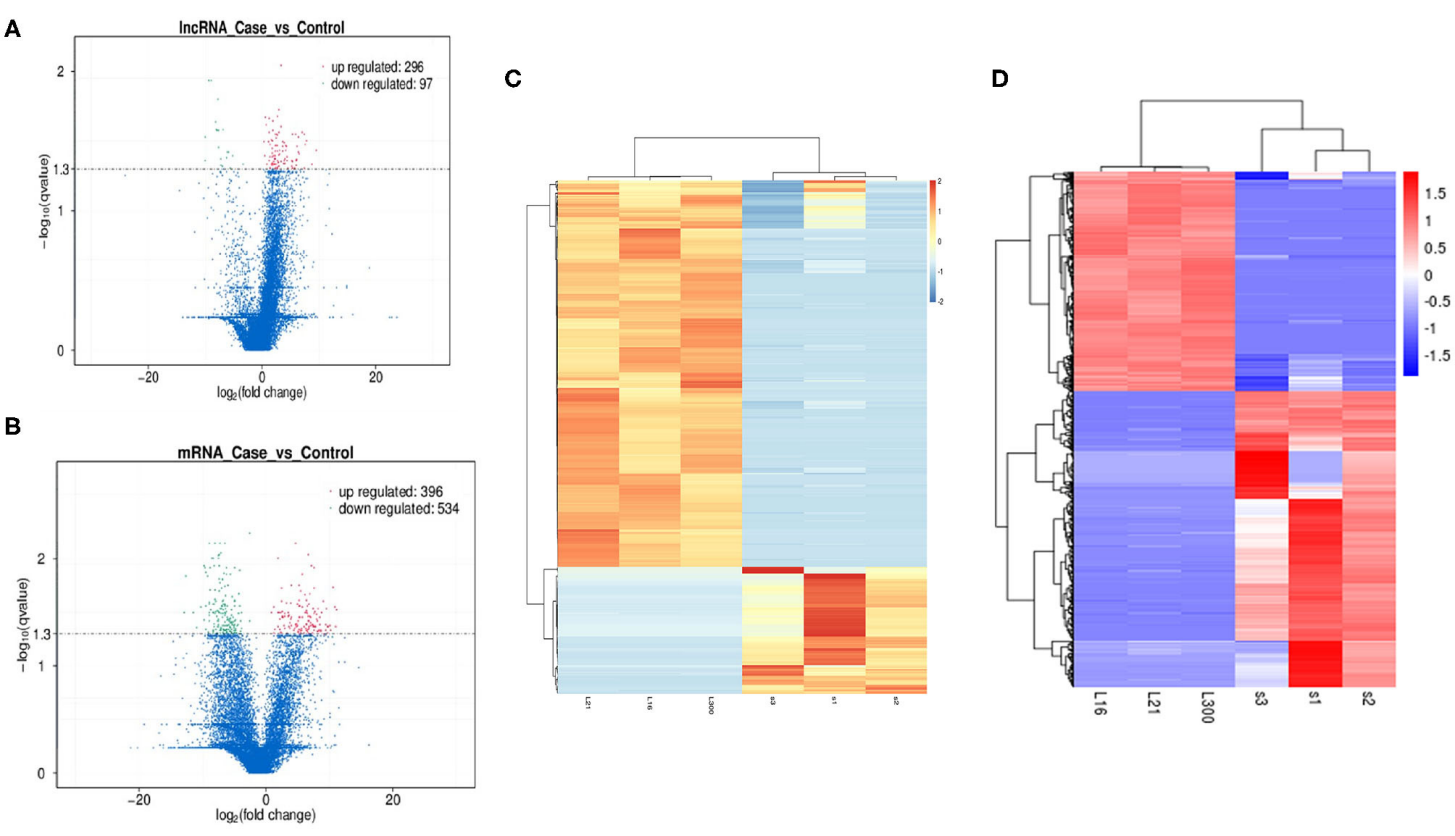
Exploration of Differentially expressed (DE) lncRNAs and mRNAs in OP vs. NC and functional analyses. **(A)** Detection of the DE lncRNAs in volcano plot. Up-regulated lncRNAs (FC ≥ 2, *P* < 0.01) are labeled red, whereas down regulated lncRNAs (FC ≤ 0.5, *P* < 0.01) are labeled green. **(B)** Detection of the DE mRNAs in volcano plot. Up-regulated lncRNAs (FC ≥ 2, *P* < 0.01) are labeled red, whereas down regulated lncRNAs (FC ≤ 0.5, *P* < 0.01) are labeled green. **(C)** Heat map analysis of DE lncRNAs in OP vs. NC. The heat map was generated by hierarchical analysis of lncRNAs and samples. The heat map was generated by hierarchical analysis of lncRNAs and samples. **(D)** Heat map analysis of DE mRNAs in OP vs. NC. The heat map was generated by hierarchical analysis of mRNAs and samples. The red and blue represented up-regulated and down-regulated transcripts, respectively.

Differential expression analysis for transcripts with *q* < 0.05 was done for both lncRNAs and mRNAs. We have identified a total of 393 differentially expressed lncRNAs in OP vs. NC, out of which 296 were up-regulated and 97 were down-regulated and are indicated in the volcano plot (Figure 4A). Similarly analysis was also done for the mRNA and we identified total of 930 mRNA transcripts were differentially regulated with 396 that were up-regulated and 534 that were downregulated (Figure 4B). Hierarchical clustering analysis showed that upregulated lncRNAs showed more relatedness to each other and down-regulated lncRNAs clustered together (Figure 4C). We also show that the pooled samples are tightly correlated to each other by using Pearson correlation coefficient method (Supplementary Figure 1). Additionally, individual samples clustered with samples within the same group as compared with samples from other group, showing high correlation among replicates of each group. Similarly, clustering was observed for the differentially expressed mRNAs within samples (Figure 4D).

**Figure 5:**
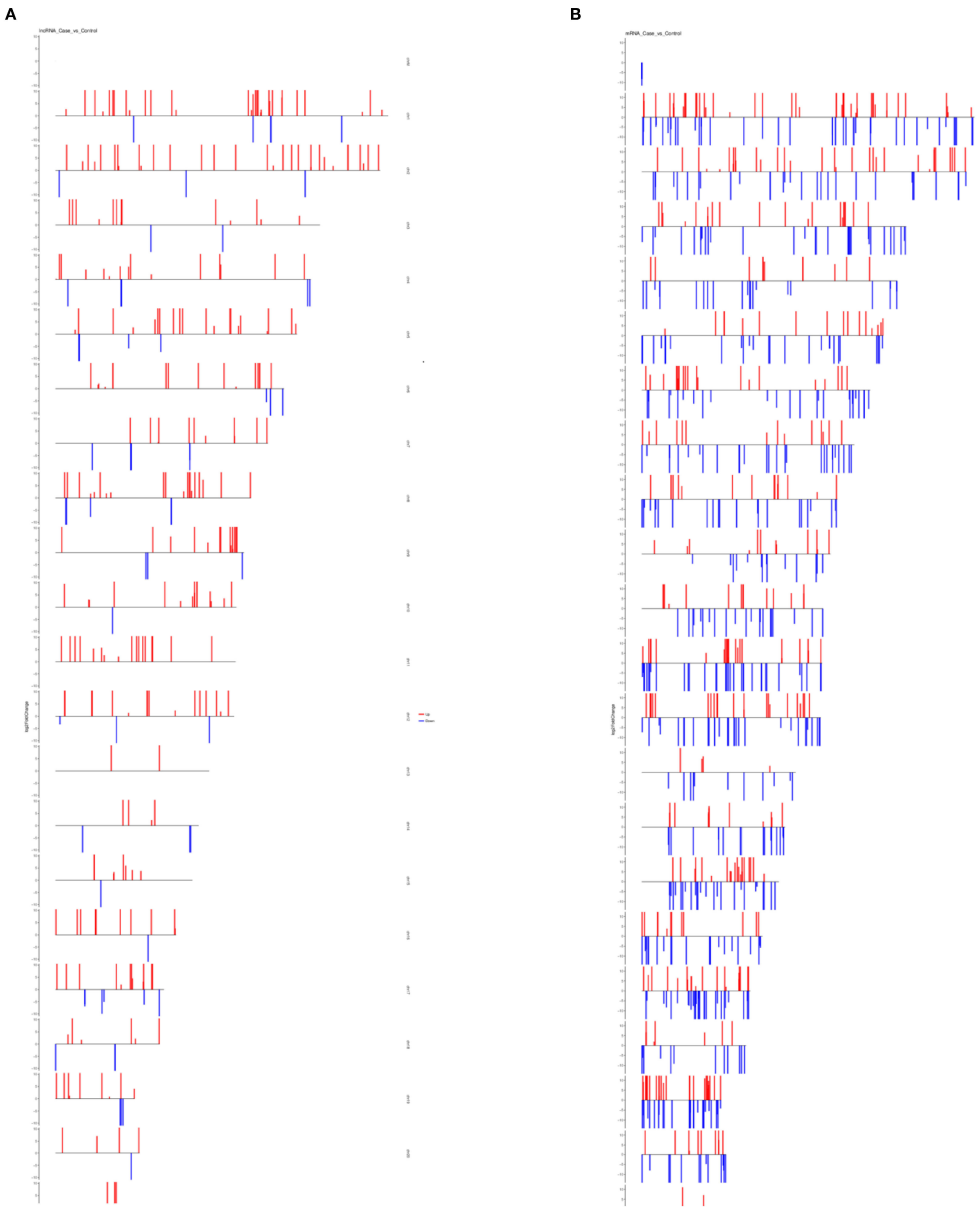
Chromosomal distribution of differentially expressed lncRNAs and mRNAs. **(A)** Distribution of differentially expressed lncRNAs on all chromosomes in the human genome. **(B)** Distribution of differentially expressed mRNAs on all chromosomes in the human genome. For both plots, red bars represent upregulated transcripts and blue bars represent downregulated transcripts.

LncRNAs are known to regulate the expression of nearby coding genes. We then studied the chromosomal distribution of differentially expressed lncRNAs and mRNAs of the human genome (Figure 5). The lncRNAs and mRNAs were not evenly distributed on the chromosomes and there was no correlation between the densities of differentially expressed mRNAs to that of differentially expressed lncRNAs.

**Figure 6:**
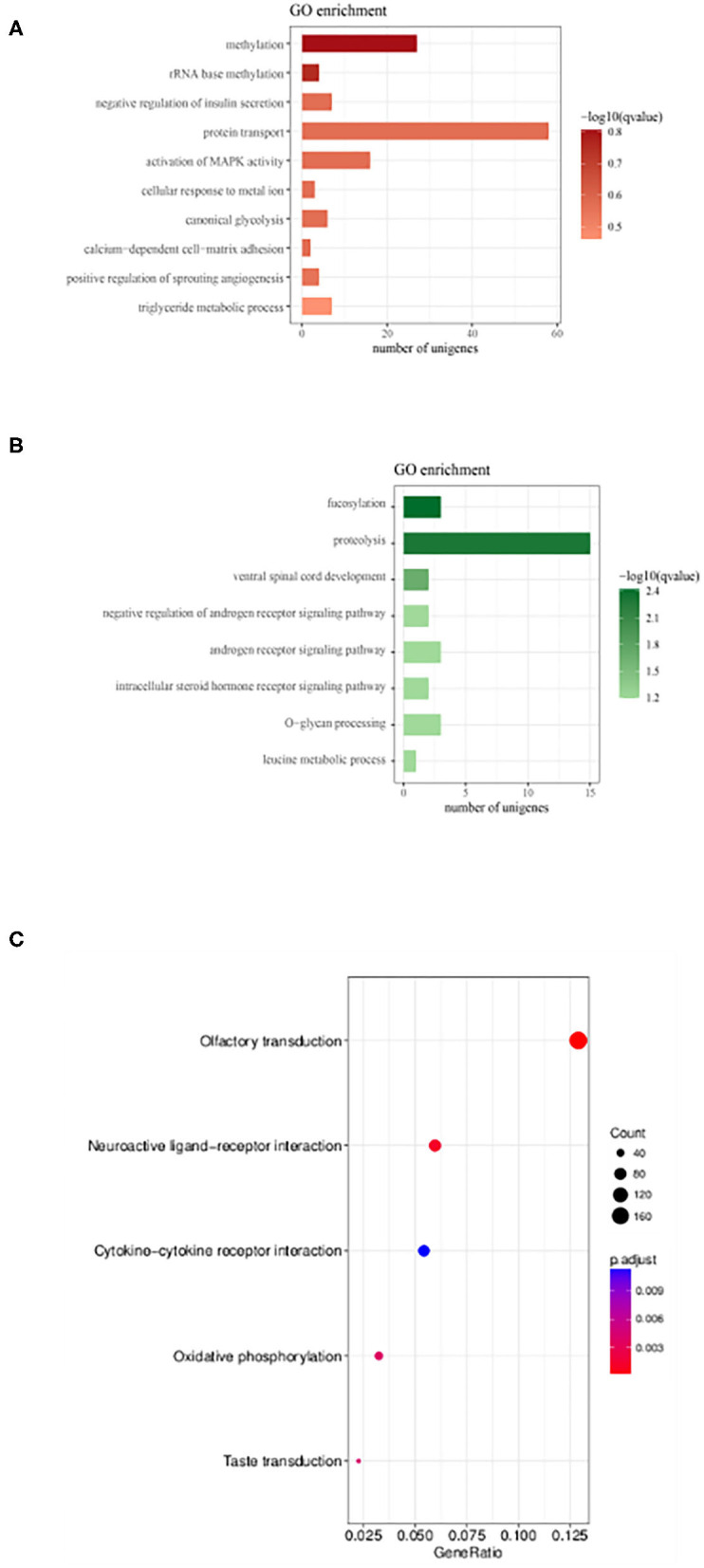
Gene Ontology enrichment and KEGG pathway analysis lncRNAs target genes. **(A,B)** GO analysis of genes which were putative regulated by up-regulated and down-regulated DE lncRNAs, respectively. Also, see Supplementary Tables 1, 2 for the list of genes. **(C)** KEGG enrichment scatter plots of differentially expressed lncRNA target genes (Supplementary Tables 3, 4).

**Figure 7:**
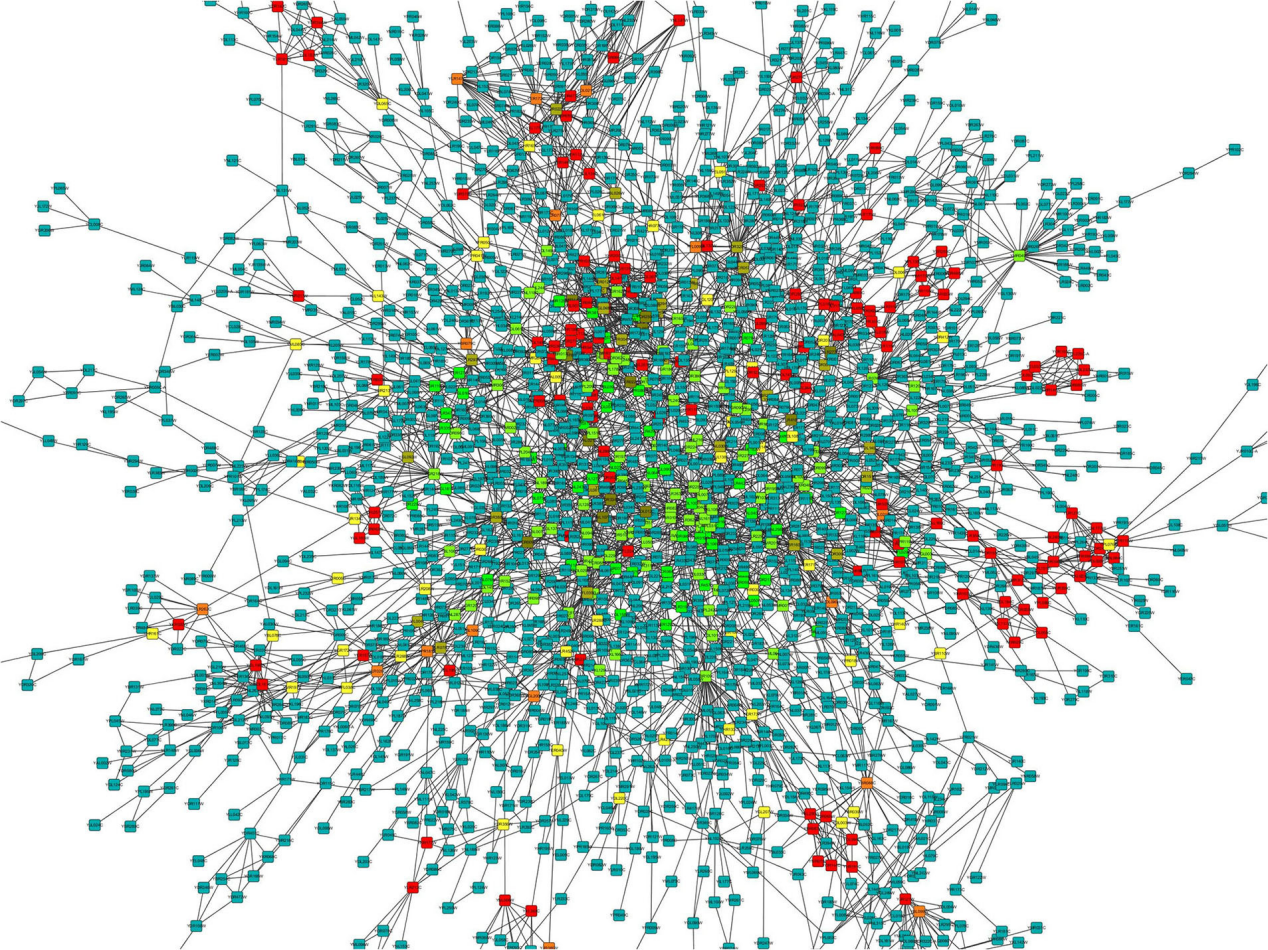
Protein-protein interaction network for differentially expressed mRNAs. The color gradient is from green to red that corresponds to the value of the clustering coefficient from low to high. The clustering coefficient indicates the connectivity between the adjacent points of this node, the higher the aggregation coefficient is, the better the connectivity between the adjacent points of this node.

To understand the physiological and molecular roles of these differentially regulated lncRNAs, gene ontology analysis (GO) (Supplementary Tables 1, 2) and KEGG pathway was performed on the target genes of these differential expressed lncRNAs (Figure 6). Bioinformatics analysis of potential target mRNAs of lncRNAs with cis-acting mechanism showed that mRNAs co-located with differentially expressed lncRNAs were highly enriched in osteoporosis-related pathways. For the upregulated lncRNAs, pathways related to protein transport, methylation and activation of MAPK activity contained the maximum number of genes (Figure 6A). GO analysis of the target mRNAs of the down-regulated lncRNAs showed genes belonging to the proteolysis, fucosylation, O-glycan processing pathways (Figure 6B). Also, see Supplementary Tables 3, 4 for the list of genes targeted by the differentially expressed lncRNAs. Protein interaction network was constructed by extracting the interaction relationship of the differentially expressed mRNAs using the STRING protein interaction database (http://string-db.org/) (Figure 7) and imported into the Cytoscape software for visualization.

### Validation of High Throughout Data With qPCR

**Figure 8:**
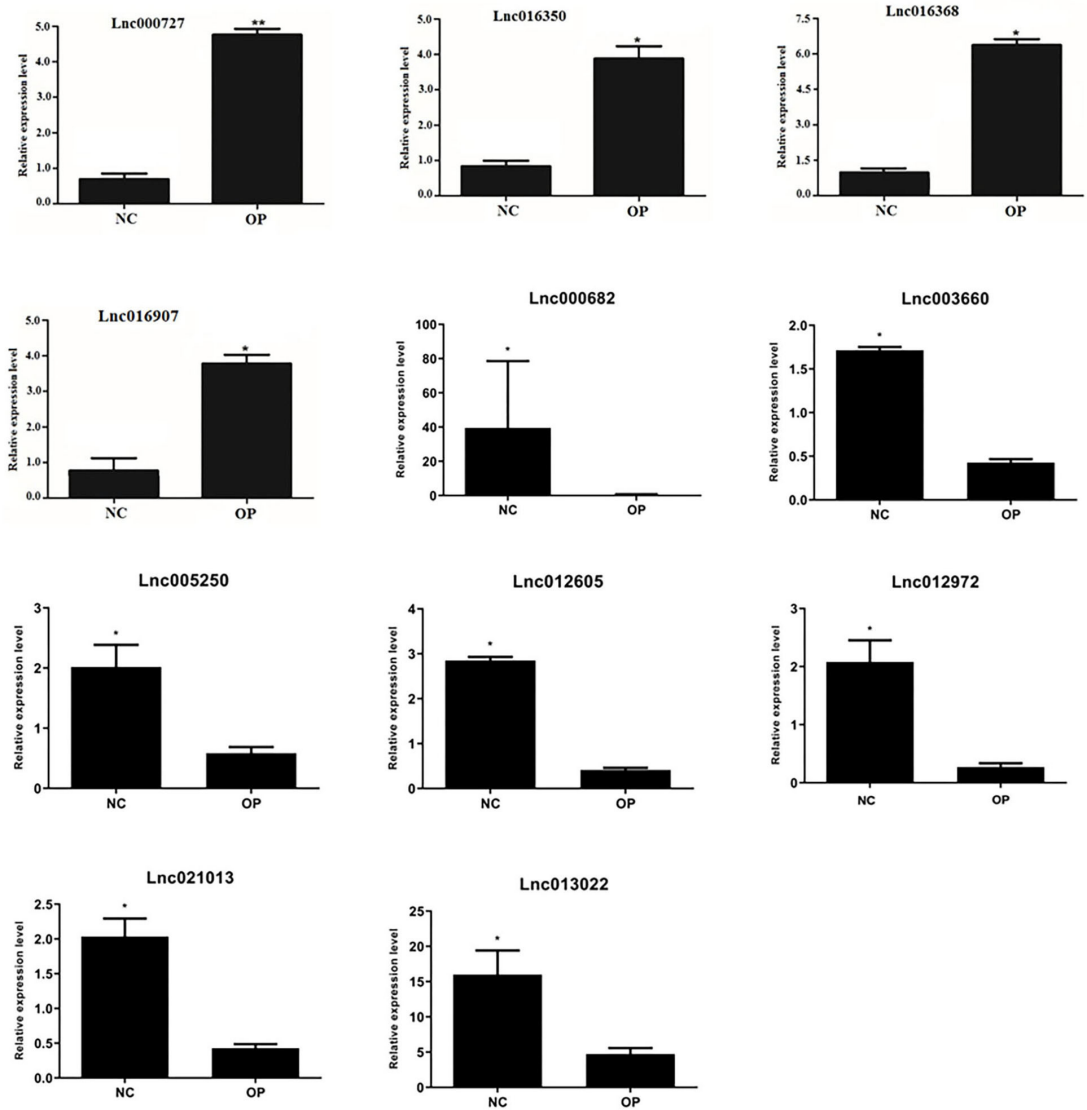
Validation of expression of lncRNA: lncRNA expression levels of four selected DE lncRNAs from RNA-seq data were validated by RT-qPCR experiment by normalizing against Actin expression level (*P < 0.05, **P < 0.01).

Differentially expressed lncRNAs obtained through RNA-sequencing and also in accordance with the proteomics data were validated by quantitative PCR analysis. qPCR validation primer details are provided in the Table 1. We analyzed 11 differentially expressed lncRNAs for qPCR validation where all the lncRNAs showed significant difference from the control levels when normalized using Actin gene (Figures 8A–K). Thus, we could successfully validate the results of the high throughput analysis by quantitative PCR analysis.

## Discussion

Aging and age related diseases are not only debilitating but are incurable due to low immunity and regenerative capacity of the elderly. One such age-related disease is osteoporosis, prevalent worldwide and affecting more than 200 million people (Reginster and Burlet, 2006). Caused by the imbalance between bone formation and bone resorption, osteoporosis leads to increased risk of hip, forearm and spine fractures. Although various treatments such as bisphosphonates, hormonal therapy, intake of calcium and Vitamin D are used for prevention of bone fractures exists, they are not effective for complete prevention (Silva et al., 2019). Early diagnosis and detection of the onset of osteoporosis can help alleviating the pain and symptoms and aid in timely medical interventions.

Recent studies have implicated the role of exosomes released by both osteoblasts and osteoclasts in regulating bone remodeling and its pathophysiology (Deng et al., 2015; Huynh et al., 2016; Sun et al., 2016). These exosomes transfer proteins and RNA such as mRNA, miRNA and other non-coding RNA to the recipient cells regulating their osteogenesis and osteo-clastogenesis (Dykes and Emanueli, 2017; Xie et al., 2017; Masaoutis and Theocharis, 2019). Developments in high throughput technology have led to the discovery of long non-coding RNAs and was soon followed by their role and association with various cellular functions and pathologic conditions (Kung et al., 2013; Dykes and Emanueli, 2017; Long et al., 2017). Even though the mechanistic details of the functional aspects of lncRNAs are not completely elucidated, reports have provided strong indication to their association with osteogenic gene regulation and its pathogenesis (Wang et al., 2015; Fei et al., 2018; Wu et al., 2018).

In this current study, we have evaluated the expression of exosomal lncRNAs in osteoporotic patients in comparison with normal individuals (Figure 1). Exosomes were isolated through ultra-centrifugation process and these were characterized by transmission electron microscopy (Figure 1C) and western blot (Figure 1B). These isolated exosomes were characterized to be membrane bound vesicles with ~100 nm in diameter. However, reconfirmation of the nanoparticle size was done using NanoSight Nano participle tracing analysis system due to the possibilities of sample dehydration and shrinkage of extracellular vesicles while processing and viewing with TEM (Bachurski et al., 2019). Nanoparticle tracking analysis reconfirmed the results obtained from TEM analysis (Figure 1D) and is a well-demonstrated procedure for determination of heterogeneity of extracellular vesicles (Gardiner et al., 2014).

Exosomes isolated from serum are known to contain lipids, proteins and RNAs such as mRNAs, miRNAs and other non-coding RNAs (Colombo et al., 2014). Specifically, of our interest, exosomes were also shown to contain lncRNAs and implicated in aging and age-related disorders (Gezer et al., 2014; Cao et al., 2019). Further, to understand the role of exosome bound lncRNAs in osteoporosis, lncRNA expression profiles of SDEs from patients with osteoporosis and normal controls (NCs) were identified and compared by high-throughput RNA sequencing. These lncRNAs were categorized and compared with the mRNAs by further analysis (Figures 2, 3).

Overall expression level of lncRNA and mRNA is observed in Figure 3D, where we see that overall expression of mRNAs was relatively significant and most of mRNAs were usually expressed at low levels. While the expression of lncRNAs were uniform between the maximum and least FPKMs (Figure 3D). Thus, it indicates that the overall expression of mRNA is varied, in contrast lncRNA levels were stable. Differential expression analysis lead to discovery of 393 differentially expressed lncRNAs in osteoporotic conditions, out of which 296 of which are upregulated and 97 are downregulated in the osteoporotic patients. The volcano plot and heat map provided sufficient evidence indicating the presence of differentially expressed lncRNAs in osteoporotic conditions (Figure 4). An earlier study on role of lncRNAs on postmenopausal osteoporosis (PMOP), identified 51 differentially expressed lncRNAs when compared with blood samples of normal controls (Fei et al., 2018). Studies on osteogenic differentiated bone marrow derived MSCs reported that more than 1,000 lncRNAs were differentially expressed when compared to non-induced control (Wang et al., 2015). After the identification of differentially expressed lncRNAs role in osteoporosis, their association with mRNAs was explored using gene ontology methods. The GO analysis identified and divided co-located or target mRNAs of the differentially expressed lncRNAs into several functional modules, related to osteoporosis pathway (Figure 6). The important lncRNAs that were differentially expressed were associated with protein transport, methylation, MAPK pathway and proteolysis. GO and KEGG analysis from a similar study reported association of differentially expressed lncRNA to mRNA in pathways related to osteoclast differentiation and MAPK signaling (Fei et al., 2018). Further, understanding of complex protein network was obtained from cytoscape software (Figure 7). Which indicates that the protein groups involved in the regulating the network are indicated with 4 colors which indicate that how the individual protein molecules are closely or widely interconnected.

Thus, we report the presence of a unique set of lncRNAs in the serum-derived exosomes with significant functional implications in osteoporosis. Although the function of the majority of lncRNAs remains to be elucidated, previous studies have indicated that lncRNAs may be involved in the pathogenesis of osteoporosis (Kung et al., 2013; Fei et al., 2018; Liu et al., 2019). We were also able to validate 11 differentially expressed lncRNAs by quantitative PCR using lncRNA specific primers (Figure 8). Identifying the key differentially expressed lncRNAs in osteoporosis not only provides support for understanding the function of lncRNAs but also contributes to developing novel biomarkers of osteoporosis. For example, an earlier report showed the presence of MALAT1 lncRNA in epithelial precursors (EPC) cell-derived exosomes. MALAT1 was shown to bind to miR-124 promoting macrophage activation and osteoclast differentiation, thus leading to bone repair (Liu et al., 2018). By profiling serum-derived exosomes for lncRNAs from osteoporotic patients, we were able to establish a non-invasive method to detect the onset of osteoporosis and further add to the understanding of the pathophysiology of osteoporosis.

## Data Availability Statement

The RNA-seq data has been deposited in NCBI Gene Expression Omnibus (GEO) under accession code GSE152293.

## Ethics Statement

The studies involving human participants were reviewed and approved by Ethics Committee of the 6th Affiliated Hospital of Kunming Medical University. The patients/participants provided their written informed consent to participate in this study.

## Author Contributions

SL: conceptualization and supervision. ZT and YZ: data curation, investigation, and project administration. XZ and YT: formal analysis. ZT, YZ, and SL: funding acquisition. XZ: software. All authors: writing—review and editing, have read, edited, and approved the current version of the manuscript.

## Conflict of Interest

The authors declare that the research was conducted in the absence of any commercial or financial relationships that could be construed as a potential conflict of interest.

## Supplementary Material

Supplementary Figure 1 Exploration of Differentially expressed (DE) lncRNAs and mRNAs in OP vs NC and functional analyses. Pearson correlation for the pooled samples, indicating the closer the correlation coefficient is to 1, the higher the similarity of expression patterns between samples.

Supplementary Table 1 GO analysis of genes targeted by upregulated lncRNAs with adjusted *p*-value.

Supplementary Table 2 GO analysis of genes targeted by downregulated lncRNAs with adjusted *p*-value.

Supplementary Table 3 GO analysis of up regulated genes targeted by differentially expressed lncRNAs.

Supplementary Table 4 GO analysis of down regulated genes targeted by differentially expressed lncRNAs.
