# Supplementary figures and images for: Osteoporosis Is Characterized by Altered Expression of Exosomal Long Non-coding RNAs
Source: Front Genet. 2020 Nov 12;11:566959. doi: 10.3389/fgene.2020.566959 (PMC7689021; doi:10.3389/fgene.2020.566959)

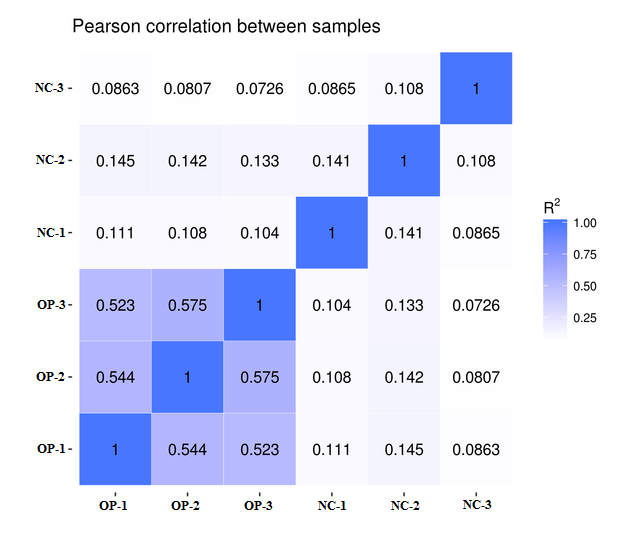

Supplement: Supplementary Figure 1 — Exploration of Differentially expressed (DE) lncRNAs and mRNAs in OP vs NC and functional analyses. Pearson correlation for the pooled samples, indicating the closer the correlation coefficient is to 1, the higher the similarity of expression patterns between samples. [file Image_1.TIF]
